# Appointment

**Published:** 1862-09

**Authors:** 


Appointment.—Dr. Mathew F. Regan of Lockport, has been appointed Surgeon of Col. McMahon's Irish Regiment. Dr. Regan has served as Assistant Surgeon of the 28th Regiment, and will bring experience and ability for the discharge of his duties.

